# An unusually rich scuttle fly fauna (Diptera, Phoridae) from north of the Arctic Circle in the Kola Peninsula, N. W. Russia

**DOI:** 10.3897/zookeys.342.5772

**Published:** 2013-10-14

**Authors:** R. H. L. Disney

**Affiliations:** 1 Department of Zoology, University of Cambridge, Downing Street, Cambridge, CB2 3EJ, U. K.

**Keywords:** Taxonomy, Phoridae, new species, Kola Peninsula

## Abstract

64 species of Phoridae, in 6 genera, are reported from the Kola Peninsula, north of the Arctic Circle. The new species *Megaselia elenae* and *Megaselia kozlovi* are described. 33 species of *Megaselia*, only known from females, are given code numbers. Keys to the species of all the females of *Megaselia* and *Phora* are provided; and also a key to the males European *Megaselia* species with a notopleural cleft.

## Introduction

The only published record of scuttle flies (Diptera: Phoridae) from the Kola Peninsula refers to *Megaselia opacicornis* Schmitz parasitizing the pupae of the leaf beetle *Chrysomela lapponica* (L.) (Chrysomelidae) (Disney et al. 2001). The town of Monchegorsk (67°55'N, 32°50'E) is situated north of the Arctic Circle. It is the centre for the smelting of copper and nickel in the Kola Peninsula and is one of the most polluted towns in the Russian Federation (Kozlov et al. 2009; Eeva et al. 2012). During 2009 and 2010 a study of the impact of this pollution on the insect fauna was undertaken (by Dr Mikhail Kozlov (University of Turku). The analysis of the results for all insect families will be reported elsewhere. For this study the samples of Phoridae were sent to me for identification. These samples included 64 species of scuttle flies, of which 3 proved to be undescribed and the females of a further 34 species of *Megaselia* can not be named until linked to their males. One new Megaselia (Disney 2011b) and a new species of *Abaristophora* are described elsewhere (Pape et al. 2013). Two new species are described below and females are characterized by means of keys to those of all the species of *Megaselia* and *Phora* obtained.

## Materials and methods

Samples were collected in 10 study sites located between 1 km North and 40 km South of Mochegorsk. In 2009 insects were collected in traps baited with dead mice, the trap being described by Ermakov (2010). In 2010 yellow traps (manufactured by Russell IPM) were employed. These traps have the inner walls of the container treated with a contact insecticide. The specimens were preserved in about 70–80% ethanol and subsequently mounted on slides in Berlese Fluid (Disney 2001). This method allows examination of gut contents and whether females are gravid, etc. The samples from the yellow traps exhibited a much higher frequency of damaged specimens than those collected in 2009.

## The species

BT refers to specimens caught in the traps baited with dead mice in 2009. 1335 scuttle flies were obtained. YT refers to those caught in the yellow traps in 2010. 344 scuttle flies were trapped. Voucher specimens, including all the type material, are deposited in the University of Cambridge, Museum of Zoology (UCMZ).

The gut contents has been noted for several specimens. Amorphous detrital material is almost certainly derived from carrion fluids.

*Abaristophora kolaensis* Disney: YT - 1 male. This species has been recently described (Pape et al. 2013).
*Anevrina thoracica* (Meigen): BT - 2 females. A new record for Russia west of the Urals.
*Anevrina unispinosa* (Zetterstedt): BT - 155 males, 140 females. The males were 11.6% of the phorids obtained in the traps baited with dead mice and the females were 10.5%. Together they were 22.1%. None were collected in the yellow traps. A new record for Russia west of the Urals. This species has been reared from dead snails (Keilin 1919) and carrion baits, including liver, dead molluscs and earthworms, and from rotting wheat flour, vegetation and mushrooms (Buck 1997, 2001).
*Megaselia albiclava* Schmitz: BT - 3 males, 10 females. YT- 1 female. None of the females were gravid. One had amorphous detrital material in the gut.
*Megaselia basseti* Disney: BT - 7 females. YT – 1 male, 13 females. The recognition of this species has recently been clarified (Disney 2011b). About half the females were gravid and some had amorphous detrital material in the gut.
*Megaselia breviterga* (Lundbeck): BT - 13 females. Some females had amorphous detrital material in the gut, which in one case included a few fungus spores. Gravid females had 5 or 7 eggs. The recognition of this species has been recently clarified (Disney 2012) and its supposed occurrence in the Nearctic Region called into question.
*Megaselia cirriventris* Schmitz: BT – 1 male. YT – 1 male. This species is prevalent in Greenland. When providing a redescription of this species Schmitz (1958: 482) erroneously synonymised *Megaselia piliventris* Schmitz (1937: 119; a replacement name for *Megaselia pilifera* Schmitz, 1936: 227) with it and then produced a hybrid description (Disney 2004). Both sexes of *Megaselia piliventris* are keyed, and critical features illustrated, by Disney (2009).
*Megaselia coccyx* Schmitz: YT - 1 male, 1 female.
*Megaselia crellini* Disney: YT – 1 male. This species belongs to a species complex previously treated as a single species (Disney 2011a).
*Megaselia eccoptomera* Schmitz: BT - 1 male, 8 females. YT - 2 males, 2 females.

### Megaselia elenae sp. n.

http://zoobank.org/788A81AD-928F-45A6-929D-9B0FCC74CE80

http://species-id.net/wiki/Megaselia_elenae

Fig. 1

#### Diagnosis.

In the key to the males of the species of the British Isles (Disney, 1989) the males will run to couplet 291. At least six other European species will run to this couplet, two of which have been described since this key. However, it belongs to a subgroup of species within those reviewed by Buck and Disney (2001). This subgroup comprises species with a bare mesopleuron, only two bristles on the notopleuron and in front of these a notopleural cleft. This subgroiup is keyed below and includes this new species.

#### Etymology.

Named after Elena Zverevra, who asked me to identify the Phoridae obtained in this study.

#### Description.

**Figure 1.**
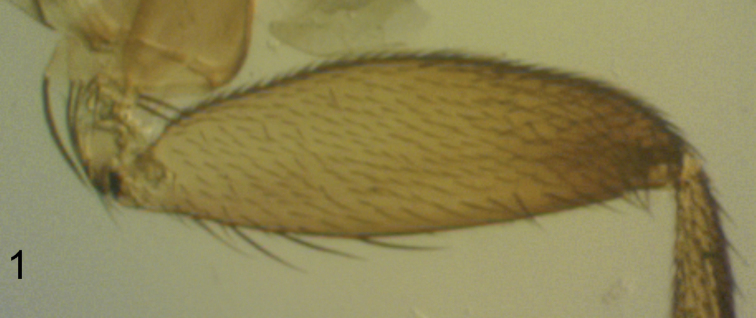
*Megaselia elenae* sp. n. male, anterior face of hind femur.

**Male.** Frons brown, clearly broader than long, with 50-60 hairs and dense but very fine microtrichia. Supra-antennal bristles (SAs) with the lower pair clearly shorter and less robust than the upper pair. [Note: the right side of frons lacks its antial bristle but has 2 pre-ocellars. The following positions of these bristles is based on the left side]. The antials slightly lower on frons than upper SAs and anterolaterals, which are about level with the latter, and about midway between upper SAs and AL bristles. Pre-ocellars slightly further apart than either is from a mediolateral bristle, which is about the same level on frons. Cheek with 4 bristles and jowl with two. The subglobose postpedicels brown, each with more than 40 subcutaneous pit sensilla (SPS) vesicles which are about 0.01 mm in diameter. Palps yellow, at most a third as broad as postpedicel but slightly longer than breadth of latter, with 6-7 bristles, the most apical being shorter than a lower SA but the longest subequal to latter, and as many hairs. Labrum yellowish brown and about half as wide as a postpedicel. Labella coloured as palps but with light brown bands on upper sides towards margins and with very few short spinules below. Thorax brown. Two notopleural bristles and a cleft in front of these, which ends just before reaching a c-shaped ridge across its path. Mesopleuron bare. Scutellum with an anterior pair of small hairs and a posterior pair of bristles. Abdominal tergites brown with small hairs except for clearly longer hairs at rear of T6. Venter brown, and with a few hairs on segments 3–6, those at rear of segment 5 being longer, and those at rear of T6 being clearly the longest. The (damaged) hypopygium is brown, with a pale brown anal tube, which is clearly longer than the length of the dorsal face of the epandrium. Each side of the latter 16–18 hairs, which are longer and stronger anteriorly but are smaller and weaker behind, and with a strong bristles (about 0.13 mm long). The hairs of the proctiger are as long and clearly thicker than hairs of the cerci. The left lobe of the hypandrium is pale grey, about 0.06 mm long, and lacking micritrichia. The right lobe is also pale grey and bare, but is only 0.02 mm long (and only 0.03 mm wide at its base). With 4 rectal papillae. Legs yellowish brown with the hind femora being the darkest and the front legs the more yellowish. Fore tarsus with a posterodorsal hair palisade on segments 1–5 and 5 a little longer than 4. Dorsal hair palisade of mid tibia extends about two thirds of its length. Hairs below basal half of hind femur clearly longer than those of anteroventral row of outer half, which are themselves long (Fig. 1). Hind tibia with 10 differentiated posterodorsal hairs and spinules of apical combs simple. Wings 1.4 mm long. Costal index 0.54 Costal ratios 3.1: 2.7: 1. Costal cilia (of section 3) 0.13–0.14 mm long. Hair at base of vein 3 as small as costal cilium at base of costa. With 3 axillary bristles, all shorter than costal cilia. Sc not reaching R1. All veins yellowish grey, with thin veins 4–6 being darkest. Membrane lightly tinged grey (just evident to naked eye when viewed against a white background). Haltere knob yellow.

#### Type material.

Holotype ♂, RUSSIA, Kola Peninsula, near Monchegorsk, in yellow trap, 26.vi–6.vii.2010, M. Kozlov (UCMZ, 17-89).

*Megaselia haraldlundi* Disney: YT – 1 female.
*Megaselia humeralis* (Zetterstedt): BT – 1 male. This species parasitizes the pupae of the leaf beetle *Chrysomela lapponica* (Chrysomelidae) (Disney and Zvereva 2008).
*Megaselia immodensior* Disney: BT – 1 male, 1 female. YT – 1 male. This species is a little variable and the specimens from the Kola Peninsula extend the range of variation a little. For example the costal index of the male is only 0.42, but in the type series it is more than 0.44 (Buck and Disney 2001). The CI of the female from the Kola Peninsula is 0.45 to 0.46.
*Megaselia fallobreviseta* Disney: BT – 3 females. YT – 5 males, 2 females. This is sibling species of *Megaselia breviseta*. The males are almost indistinguishable but the females of the two species are readily distinguished (Disney 2011b). It has been reared from the caterpillar tents of an ermine moth (*Yponomeuta* sp.) in Germany.
*Megaselia fuscovariana* (Schmitz): BT – 1 male, 1 female. YT - 1 male, 1 female.

### Megaselia kozlovi sp. n.

http://zoobank.org/33869855-A189-4460-B780-E546125885AD

http://species-id.net/wiki/Megaselia_kozlovi

Figs 3 –7

#### Diagnosis.

In the key to the males of the species of the British Isles (Disney 1989) the males will run to couplet 245, Lead 1, where one is directed to return to couplet 241. It runs out there to Lead 1, but the species of this lead is covered by a revision of a group of species (Buck and Disney 2001) from which the new species is immediately excluded by having 3, not 2, bristles on the notopleuron. Furthermore, the small anterior pair of hairs on the scutellum contrast with the bristle like anterior scutellars of the species it most resembles in this complex.

#### Etymology.

Named for its collector Mikhail Kozlov.

#### Description.

**Figures 2–3.**
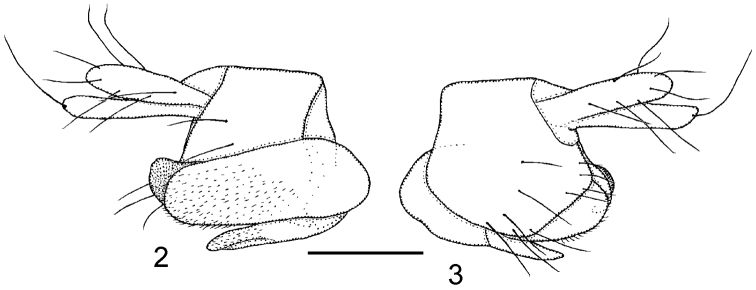
*Megaselia kozlovi* sp. n. male, hypopygium. **2** right face **3** left face. Scale line: 0.1 mm.

**Male.** Frons brown, clearly broader than long, with 30–42 hairs and very dense but very fine microtrichia. Supra-antennal bristles (SAs) unequal, the lower pair being about as long as the longest (apical) bristle of the palp but less robust than it. The antials lower on frons than anterolaterals, and more than twice as far from upper SAs as either is from an AL bristle. Pre-ocellars slightly closer together than either is from a mediolateral bristle, which is very slightly higher on frons. Cheek with 2–3 short bristles and jowl with one long and one short bristle. The subglobose postpedicels brown, without SPS vesicles. Palps yellow, at most a third as broad as postpedicel but about 1.4 times as long as breadth of latter, with 5–8 bristles, 3–4 being long and the rest short, and 1–3 hairs. Labrum pale yellow and about 0.8 times as wide as a postpedicel. Labella a little paler than palps, with only a few short spinules below but with several pale teeth along their inner edges.. Thorax brown. Three notopleural bristles and no cleft in front of these. Mesopleuron bare. Scutellum with an anterior pair of small hairs and a posterior pair of bristles. Abdominal tergites brown with T6 being longest and with longer hairs at its rear margin than on the rest of the tergites. Venter grey, and with fine hairs on segments 3–6. Hypopygium brown, with a light brown anal tube, and as Figs 2–3. Legs with yellowish brown hind femora and otherwise dusky yellow (apart from the largely brown mid coxae). Fore tarsus with segments 1–3 somewhat stout and with at least one row of hairs below each reduced to short pale spinules. A posterodorsal hair palisade on segments 1–4 and 5 about as long or slightly shorter than 4. Dorsal hair palisade of mid tibia extends about two thirds its length. Hairs below basal half of hind femur longer than those of anteroventral row of outer half. Hind tibia with 11–12 differentiated posterodorsal hairs and spinules of apical combs simple. Wings 1.3–1.4 mm long. Costal index 0.48–0.50. Costal ratios 3.0–3.6: 2.0–2.4: 1. Costal cilia (of section 3) 0.07–0.08 mm long. No hair at base of vein 3. With 2 axillary bristles, the outer being a little longer than costal cilia. Sc not reaching R1. Thick veins and vein 7 yellowish grey, thin veins 4–6 grey. Membrane tinged grey (evident to naked eye when viewed against a white background). Haltere knob yellow.

**Figure 4.**
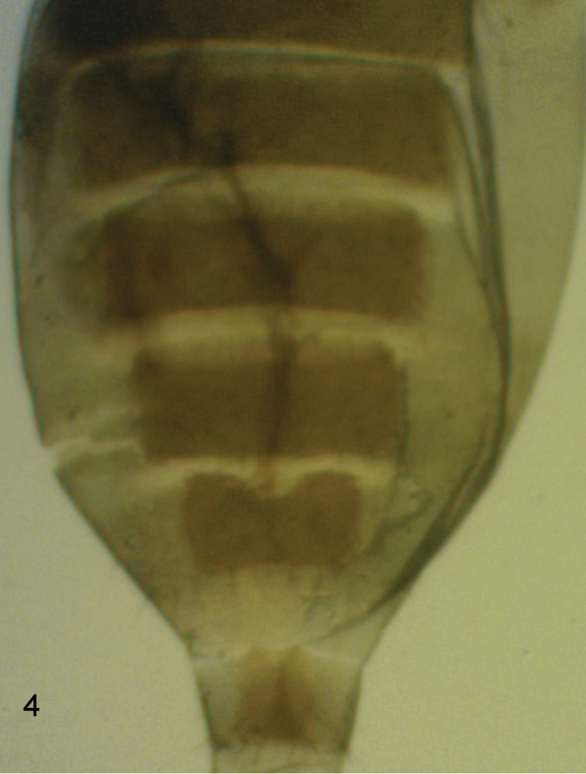
*Megaselia kozlovi* sp. n. female, abdominal tergites 3–7.

**Figure 5.**
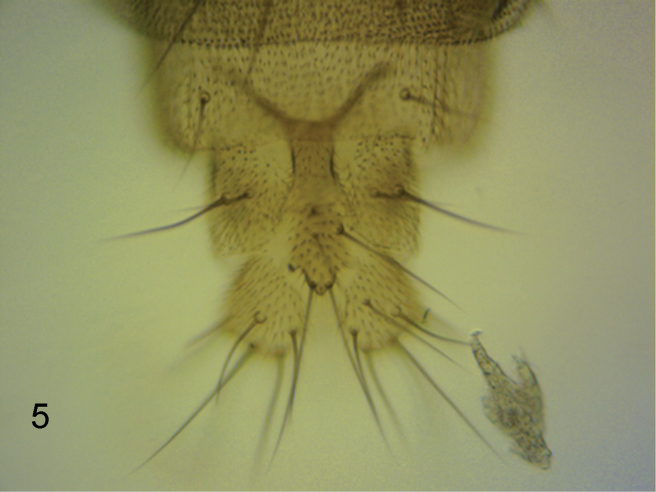
*Megaselia kozlovi* sp. n. female, tip of abdomen from above.

**Figure 6.**
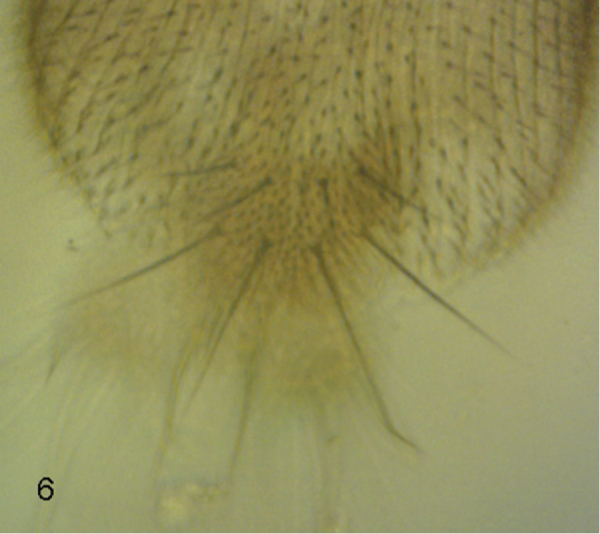
*Megaselia kozlovi* sp. n. female, lobe at rear of abdominal sternite 8.

**Figure 7.**
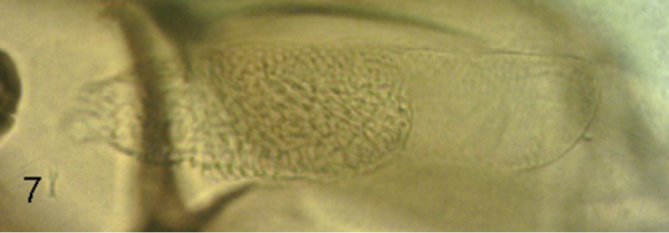
*Megaselia kozlovi* sp. n. female, Dufour’s crop mechanism (anterior end to left).

**Female.** Head similar to male except palps with 7–8 bristles, the longer ones being a little shorter than those of male, and with 3–7 hairs. Thorax as male. Abdominal tergites brown. T3-T7 as Fig. 4. Venter grey, and with hairs below segments 3–6. Sternite 7 an isosceles triangle tapering to an anterior point and with 4 longer hairs at its straight hind margin and at least as many smaller hairs further forward. The single lobe at rear of sternum 8 as Fig. 6. Cerci and epiproct as Fig. 5. With 4 rectal papillae. Furca not evident. Dufour’s crop mechanism as Fig. 7. Legs similar to male except the front tarsus has segment 1–3 as slender as the rest, segment 5 is a little longer than 4 and 5 may or may not have a posterodorsal hair palisade. Wing as male except 1.4–1.6 mm long. Costal index 0.47–0.49. Costal ratios 2.8–3.9: 1.5–2.5: 1. Costal cilia 0.08–0.09 mm long. Otherwise it and haltere as male. Two females were gravid, one with 5 eggs and the other with 6. These eggs measured 0.3–0.4 mm long and 0.16–0.17 mm wide.

#### Type material.

Holotype ♂, RUSSIA, Kola Peninsula, near Monchegorsk, at dead mouse, 18–25.vii 2009, M. Kozlov (UCMZ, 17-40). Paratypes, 2F the same except (17-48 & 72), 1 F, 25.vii–1.viii.2009 (UCMZ, 17-44), 1 male, in yellow trap, 6-16.vii.2010 (UCMZ, 17-83).

*Megaselia limburgensis* (Schmitz): YT – 1 male.
*Megaselia nudiventris* (Wood): YT – 1 male. This species has recently been rescued from synonymy (Disney 2011a).
*Megaselia parnassia* Disney: BT – 1 male, 10 females. YT – 2 males. A gravid female had 4 eggs (HF = 0.8-0.9 mm long). Some females had amorphous detrital material in the gut, which in two cases included several fungus spores. This is mainly a boreo-alpine species of northern Europe and Canada.
*Megaselia petraea* Schmitz: BT – 2 males, 12 females. One female was gravid.
*Megaselia sordida* (Zeterstedt): BT – 744 females, which is 56.3% of the phorids obtained in the traps baited with dead mice. This was the commonest species in the traps. YT – 12 males, 74 females. Thus the males were 3.4% of the phorids obtained in yellow traps and the females were 20.8%. Only a few had amorphous detrital material in the gut. Only a few were gravid. Of these one had 19 eggs, which measured 0.67 mm long and 0.25 mm wide (HF = 1.02 mm long). One had 32 half developed eggs (HF = 0.99 mm long).

The following species are only known in the female sex and are given code numbers only until they can be linked to their males. The sequence of numbers is incomplete as some females were subsequently linked to their males.

*Megaselia* species 3: BT – 25 females. YT – 3 females. None were gravid but more than 40 immature eggs were recorded. There was no evidence of feeding on carrion fluids.
*Megaselia* species 4: YT – 1 female.
*Megaselia* species 5: BT – 30 females. These were 2.2% of phorids caught at dead mice. YT – 94 females, which was 27.3% of the phorids in the yellow traps. None were gravid and one had amorphous detrital material in the gut.
*Megaselia* species 6: BT – 6 females. One with 2 relatively large eggs, that were 0.74 mm long and 0.28 mm wide (HF = 0.91 mm long). There was no evidence of feeding on carrion fluids.
*Megaselia* species 7: BT – 2 females. None was gravid and there was no evidence of feeding on carrion fluids.
*Megaselia* species 8: BT – 11 females. YT – 8 females. None were gravid and there was no evidence of feeding on carrion fluids.
*Megaselia* species 10: BT – 7 females. YT – 1 female. None were gravid but one had fungus spores in the crop.
*Megaselia* species 11: BT – 1 female. With amorphous detrital material in the gut.
*Megaselia* species 12: BT – 1 female. With amorphous detrital material in the gut.
*Megaselia* species 13: BT – 1 female.
*Megaselia* species 16: BT – 29 females. YT – 7 females. Most had amorphous detrital material in the gut and in one case this included short, thick walled, fungus bodies. One had 2 mature eggs remaining, the rest of the batch evidently having been deposited.
*Megaselia* species 17: BT – 2 females.
*Megaselia* species 18: BT – 5 females. Three with amorphous detrital material in the gut, one of which included a few spindle-shaped fungus spores.
*Megaselia* species 19: BT – 20 females. Three with amorphous detrital material plus fungus spores in the gut, the spores being round in one case and spindle shaped in two. A few pollen grains were present in guts of two specimens. One with 24 half developed eggs.
*Megaselia* species 20: BT – 3 females.
*Megaselia* species 21: BT – 2 females.
*Megaselia* species 24: BT – 1 female, with dark amorphous material in the gut.
*Megaselia* species 25: BT – 1 female.
*Megaselia* species 26: BT – 1 female.
*Megaselia* species 27: BT – 1 female. With amorphous detrital material in the gut.
*Megaselia* species 28: BT - 1 female.
*Megaselia* species 29: BT – 1 female. With amorphous detrital material in the gut.
*Megaselia* species 30: BT – 1 female. With pollen grains in gut.
*Megaselia* species 31: BT – 2 females. One was gravid, with 8 eggs which measured 0.51 long and 0. 25-0.26 mm wide (HF = 0.90 mm long). The other with amorphous detrital material in the gut.
*Megaselia* species 33: BT – 2 females. One had a single egg remaining. It measured 0.32 mm long and 0.12 mm wide (HF = 0.49 mm long).
*Megaselia* species 34: BT – 3 females. One had two eggs remaining, they measured 0.51-0.54 mm long and 0.21-0.23 mm wide (HF = 0.73 mm long). Another had granular material in the gut.
*Megaselia* species 36: BT – 1 female. With a little amorphous detrital material in the gut.
*Megaselia* species 37: BT – 1 female. YT – 10 females.
*Megaselia* species 38: BT – 1 female.
*Megaselia* species 39: BT – 1 female.
*Megaselia* species 40: BT – 1 female. The gut had fine amorphous debris. Fungus mycelium was present in the abdomen.
*Megaselia* species 42: BT – 2 females. Both had fungus spores in the gut. One was gravid. The eggs are 0.99-1.00 mm long and 0.32 mm wide and have a plastron running the length of the dorsal face. There were 14 eggs (HF = 1.21 mm long).
*Megaselia* species 46: BT – 1 female.
*Megaselia* species 47: BT – 1 female. There was a single relatively large egg measuring 0.66 mm long and 0.29 mm wide; this egg being longer than the length of the hind femur (at 0.47 mm).
*Microselia forsiusi* (Schmitz): BT – 1 female. This species was previously only known from Finland.

### Concerning the genus *Phora*

The recognition of the species in this genus is based on the males, with particular attention to the hypopygium. In his keys to the Palaearctic species, Schmitz (1953, 1955) divided the species into three groups. Section I species have 2 or 3 anterior bristles on the basal half of the hind tibia. Those of Section II have only 1 such bristle on the hind tibia, and 2 anterior bristles on the basal half of the mid tibia. The species of Section III have only 1 of each of such bristles on the mid and hind tibiae. However, *Phora dubia* in Section I (under a synonym, *loc. cit.*, 1955: 342) has 1 or 2 bristles on its hind tibia, and in some specimens there is 1 on one leg and 2 on the other. Likewise *Phora stictica* in Section II occasionally has 2 bristles on the hind tibia and only 1 bristle on the mid tibia; and *Phora artifrons* in the same section likewise sometimes has only 1 bristle on the mid tibia. Such variation has to be taken into account when trying to match up females with their males. In addition there is evidently some sexual dimorphism in these bristle number differences. For example for some *Phora atra* (Meigen) males I have obtained mating there were 4 or 5 dorsal bristles on the mid tibia, but only 2 or 3 on those of their female partners. Furthermore, some males of this species occasionally have 2 anterior bristles on at least one mid tibia; which means they would be in Schmitz’s Section II instead of Section III. One specimen attributed to *Phora holosericea* has no anterior bristles on the mid tibiae. Other variation occurs with respect to colour, such as the costa, the thin veins and the wing membrane (including the extent of the regions devoid of microtrichia), and the front tibia and tarsus. As none of the females were gravid, apart from one with immature eggs, some of the paler specimens were probably only recently emerged from their pupae and were not fully darkened. The result of these variations is that females are still poorly known in this genus. However, Cook and Mostovski (2002) have shown the use of 16S mitochondrial sequences in linking unknown females to their correct males, and then correlating these molecular signatures with small morphological differences. In the collections from the Kola Peninsula only five species represented by males were obtained. I have therefore identified the females on the assumption that only these five species needed to be considered. A tentative key to the species of females from the Kola Peninsula is given below.

*Phora artifrons* Schmitz: YT – 1 male.
*Phora dubia* (Zetterstedt): BT – 2 females. YT – 1 male, 2 females.
*Phora holosericea* Schmitz: BT – 41 females, which was 3.1% of the phorids caught at dead mice. YT – 4 males, 83 females. Thus the females were 23.4% of the phorids caught in the yellow traps. Larvae prey upon the root feeding Aphididae and Pemphigidae (Yarkulov, 1972).
*Phora pubipes* Schmitz: BT – 5 females. YT – 3 males, 1 female. One female had 20 immature eggs (HF = 1.9-2.0 mm long).
*Phora stictica* Meigen: YT – 2 males, 11 females. These represented 3.7% of the phorids caught in the yellow traps.
*Triphleba palposa* (Zetterstedt): Bt – 1 female.

**Figure 8.**
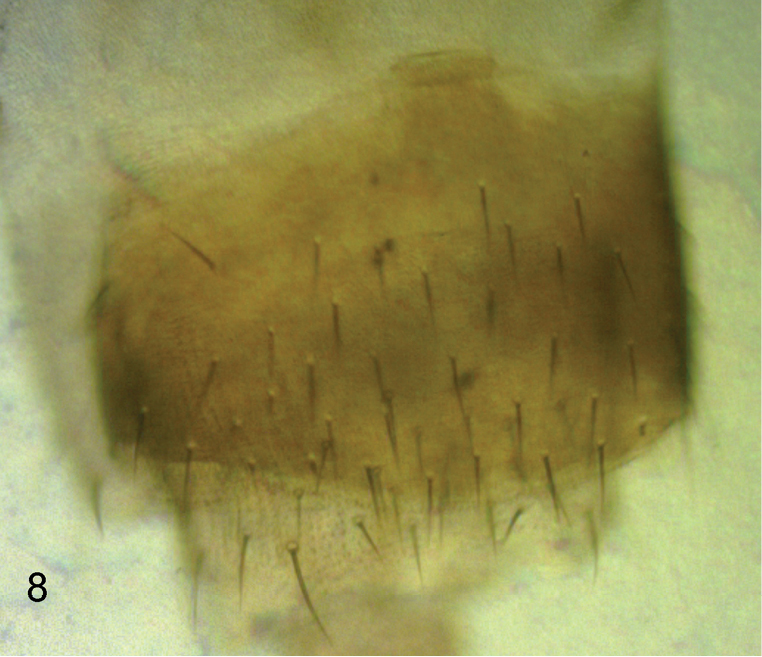
*Triphleba palposa* female, abdominal sternite 7.

**Figure 9.**
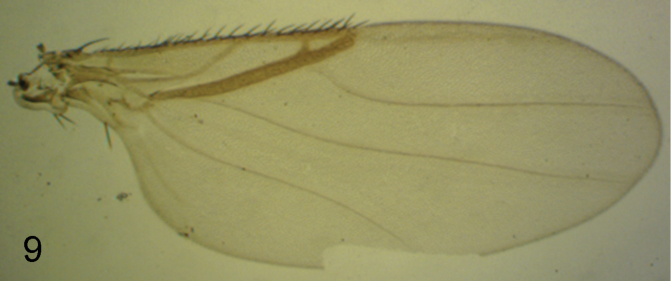
*Triphleba palposa* female, right wing.

The female of this species was only briefly described by Schmitz (1943) and without any figures. The abdominal sternite 7 was described thus “anscheinend gross, an den Seiten weit hinaufreichend”. Figs 8–9 illustrate this.

*Triphleba renidens* Schmitz: YT – 1 female.

### Key to *Megaselia* females of species recorded in the Kola Peninsula

This key provides a preliminary sorting only. Identification requires checking out the details given under the brief description of each species.

Note: Variable species are keyed both ways at several couplets

**Table:** 

1	Mesopleuron with hairs and sometimes with differentiated bristles near rear margin	2
–	Mesopleuron bare	32
2	With three bristles on the notopleuron	3
–	With two bristles on the notopleuron. (Vein Sc runs into vein 1 and fuses with it. Abdominal tergite 3 with concave hind margin and shorter than both T2 and T4. (Hind femora yellow. Haltere knobs yellow. Scutellum with an anterior pair of hairs and a posterior pair of bristles. Mesopleuron with hairs only)	Species, 30
3	Vein Sc runs into vein 1 (R1) and fuses with it	4
–	Vein Sc ends before reaching vein 1	5
4	Scutellum with two pairs of robust bristles, but the anterior pair are shorter than those behind. Palps dusky yellow or brown	5
–	Scutellum with an anterior pair of hairs and a posterior pair of bristles. Palps clear yellow	Species, 24
5	Haltere knob brown or greyish brown	6
–	Haltere knob yellow	20
6	Mesopleuron in addition to hairs with one or more short bristles near hind margin	7
–	Mesopleuron with hairs only	8
7	Scutellum with two pairs of robust bristles, but the anterior pair are shorter than those behind. Palps yellow	*Megaselia basseti* Disney
–	Scutellum with an anterior pair of hairs and a posterior pair of bristles Palps brown	8
8	Palps yellowish brown to brown	9
–	Palps yellow	10
9	Ventral edge of hind femur slightly concave just beyond base. Wings distinctly grey when viewed against a white background	*Megaselia eccoptomera* Schmitz
–	Ventral edge of hind femur straight. Wing only faintly grey when viewed against a white background	10
10	Hind femora yellow	11
–	Hind femora light brown or darker	12
11	Scutellum with two pairs of robust bristles, but the anterior pair are shorter than those behind. Cerci about 3× as long as broad	20
–	Scutellum with an anterior pair of hairs and a posterior pair of bristles. Cerci less than twice as long as broad	Species, 21
12	Base of vein 3 with a hair, which is sometimes minute	13
–	No hair at base of vein 3	18
13	Scutellum with an anterior pair of hairs and a posterior pair of bristles	14
–	Scutellum with two pairs of robust bristles, but the anterior pair are shorter than those behind	20
14	The hairs below basal half of hind femur about as long or shorter than those of anteroventral row in outer half	15
–	Some of these hairs clearly longer than hairs of anteroventral row in outer half	Species, 49
15	The lower faces of labella with only a few short spinules	16
–	Lower faces of labella with numerous short spinules (at least 40 on each)	Species, 36
16	The costal cilia of section 3 clearly longer than outermost axillary bristle	17
–	The costal cilia of section 3 about as long as outermost axillary bristle	20
17	Costa less than half length of wing	18
–	Costa more than half length of wing	20
18	Mesopleuron with hairs only gradually increasing in size towards rear margin	19
–	Mesopleuron with small hairs contrasting strongly with the single bristle at rear margin. (Abdominal tergites 5 to 8 as Fig. 10)	*Megaselia cirriventris* Schmitz
19	With more than 3 bristles on axillary ridge of wing. Labrum brown and wider than diameter of postpedicel	Species, 5
–	With only 2 axillary bristles. Labrum pale yellow and not as wide as postpedicel	Species, 13
20	Scutellum with two pairs of robust bristles, but the anterior pair are shorter than those behind	1
–	Scutellum with an anterior pair of hairs and a posterior pair of bristles (or rarely with a single pair of bristles only)	23
21	Wing more than 2.5 mm long and costa extends at least half the length of wing	22
–	Wing less than 2.5 mm long and costa less than half length of wing	24
22	Hind femora yellow with brown tips and hairs below basal halves clearly longer than those of anteroventral rows in outer halves	Species, 39
–	Hind femora pale chestnut brown and the hairs below basal halves clearly shorter than those of anteroventral rows in outer halves	Species, 16
23	Hind femora yellow or yellow with brown tips, or shading to brown in outer half	24
–	Hind femora uniformly brown	26
	Note. Variable species are keyed both ways.
24	Hind femora with at least their tips brown. Axillary ridge with at least four bristles. Vein 3 with a hair at base, but it may be very small (and occasionally absent)	25
–	Hind femora entirely yellow. Axillary ridge of wing with fewer than four bristles. No hair at base of vein 3. (Postpedicels yellowish grey. Labella large, pale and with numerous microtrichia and small spinules below)	Species, 34
25	With at most 6 axillary bristles. Hairs below basal halves of hind femora shorter that those in anteroventral rows of outer halves	26
–	With more than 6 axillary bristles. Hairs below basal halves of hind femora about as long as those in anteroventral rows of outer halves	Species, 26
26	Lower faces and apical lateral regions of labella with relatively few short spinules (less than 20 below each)	27
–	Lower faces and apical lateral regions of labella with numerous short spinules (at least 30 on each)	29
27	Hind femora entirely brown	28
–	Hind femora yellow with brown tips. (Postpedicels with SPS vesicles. Labrum yellow. DCM with narrow posterior region)	*Megaselia fuscovariana* (Schmitz)
28	Hairs below basal half of hind femur shorter than those of anteroventral row of outer half. Front femora usually almost as dark as mid and hind femora. Labrum typically chestnut brown. Wings lightly tinged grey. DCM narrow, the width being at most a quarter of the length	*Megaselia sordida* (Ztterstedt)
–	Hairs below basal half of hind femur at least as long as those of anteroventral row of outer half. Front femora usually distinctly more yellowish brown than mid and hind femora. Labrum yellow to yellowish brown. Wings darker and more yellowish grey. DCM broader, its width being about half its length	*Megaselia albiclava* Schmitz
29	All femora dark brown. Lower supra-antennal bristles (SAs) longer and more robust than bristles on palps	30
–	Front and middle femora more yellowish brown or paler. Lower SAs shorter and less robust than bristles on palps	31
30	Palps clear yellow. Abdominal tergite 7 broader than long	*Megaselia coccyx* Schmitz
–	Palps yellowish brown. T7 clearly longer than broad	Species 37
31	Costal cilia (of section 3) less than 0.12 mm long. Sc almost always reaching vein 1	*Megaselia limburgensis* (Schmitz)
–	Costal cilia more than 0.12 mm long. Sc clearly ending before vein 1	Species, 8
32	Vein Sc runs into vein 1 (R1) and fuses with it	33
–	Vein Sc ends before reaching vein 1	45
33	Hind femora brown and haltere knob brown or if a little yellowish the hind femora are clearly brown	34
–	Hind femora yellowish brown to yellow with brown tip and haltere knob yellow, but when a little dusky the hind femora are mainly yellow	40
34	Notopleuron with three bristles	35
–	Notopleuron with only two bristles	36
35	Scutellum with an anterior pair of hairs (subequal to those near rear of scutum) and a posterior pair of robust bristles. Abdominal tergite 2, apart from anterolateral processes, clearly narrower than T1 and with a concave hind margin. T3 with its front margin narrower than its hind margin, T7 a Y shape and clearly narrower than T6	Species, 11
–	Scutellum with four robust bristles. T2 a little wider than T1 and with a straight hind margin. T3 with front margin slightly wider than hind margin. T7 broader than long and wider than T6. (T6 is longer than broad)	Species, 42
36	Scutellum with four robust bristles	37
–	Scutellum with a posterior pair of robust bristles and an anterior pair of hairs (at most subequal to those near rear of scutum or rarely absent)	38
37	Abdominal tergite 6 longer than greatest breadth and its rear margin is narrower than the front margin of T7. The outermost axillary bristles are longer than the costal cilia of costal section 3. DCM rounded behind	Species, 42
–	T6 clearly broader than long and wider than the narrow T7. The outermost axillary bristles are shorter than the costal cilia of section 3. DCM bilobed behind	Species, 38
38	Cerci at least 2.5× as long as broad. Lobes at rear of abdominal sternum 8 short and broader than long	39
–	Cerci at most twice as long as broad. The lobes at rear of S8 at least 3× as long as broad	Species, 12
39	Abdominal tergite 7 broad, its greatest width being about half that of rear margin of T6	Species, 46
–	T7 narrow, being at its widest at most only a third as wide as rear margin of T6	*Megaselia petraea* Schmitz
40	Scutellum with a posterior pair of robust bristles and an anterior pair of hairs (at most subequal to those near rear of scutum or rarely absent)	41
–	Scutellum with four robust bristles. (The lobes at rear of S8 taper to a point. Abdominal tergite 4 not as wide as front margin of 3)	Species, 28
41	Wing more than 1.6 mm long	42
–	Wing less than 1.5 mm long (its membrane pale and likewise veins 4–6)	45
42	Abdominal tergite 3 with a more-or-less straight hind margin and at least as long as T4	43
–	T3 with a concave hind margin and typically shorter than T4 (Fig. 10). (Dufour’s crop mechanism as Fig. 11)	*Megaselia breviterga* (Lundbeck)
43	Cerci with rounded tips. Hypoproct with small denticles as well as the larger microtrichia	44
–	Cerci tapered towards tips. Hypoproct lacks small denticles. (S8 lobes symmetrical so that their rounded tips are directed rearwards)	Species, 7
44	Hairs below basal half of hind femur clearly longer than adjacent hairs of anterior face. Postpedicels with SPS vesicles. (Lobes of abdominal sternite 8 asymmetrical and with the sides longer than the inner edges, so that the tips are inclined towards the midline)	Species, 19
–	Hairs below basal half of hind femur about as long or only slightly longer than adjacent hairs of anterior face. Postpedicels without SPS vesicles	Species, 4
45	Scutellum with four robust bristles, but the anterior pair may be a little shorter and less robust than those behind	46
–	Scutellum with a posterior pair of robust bristles and an anterior pair of hairs (at most subequal to those near rear of scutum or rarely absent)	54
46	Abdominal sternite 7 a narrow bar that is narrowest at the rear end where it bears a single bristle. The lobes at the rear of sternum 8 are rounded with dusky bare rims beyond the bristles	Species, 20
–	Without this combination	47
47	All femora yellow or dusky yellow apart from brown tips to hind femora	48
–	All femora essentially brown to dark brown	51
48	Abdominal tergite 6 longer than broad (Fig. 20). Posterodorsals of hind tibia strongly differentiated	49
–	Not so	50
49	Labrum massive (Fig. 21). Postpedicels yellow at base, darker apically and without SPS vesicles. Vein 3 with 1–2 hairs at base. Hairs below basal half of hind femur shorter than those of the anteroventral row in the outer half	*Megaselia humeralis* (Zetterstedt)
–	Labrum less massive (e.g. Fig. 22). Postpedicels uniformly brown and with SPS vesicles. Vein 3 with minute hair (shorter than width of vein) or without hair. Hairs below hind femur longer than those of a-v row of outer half	51
50	With more than 30 hairs on segments 3 to 5 of abdominal venter as well as many on 6, which has longer hairs at rear margin. Furca not evident. Lobes at rear of sternum 8 as Fig. 18. (Costal index exceeds 0.46. Costal cilia of section 3 at least 0.10 mm long)	*Megaselia haraldlundi* Disney
–	With less than 15 hairs on segments 3–5 of venter. A strongly sclerotised furca present (Fig. 25). Lobes at rear of abdominal sternum 8 rounded as Fig. 24	Species, 6
51	Abdominal tergite 7 as Fig. 14. Sternite 7 as Fig. 15. Lobes at rear of S8 and hypoproct as Fig. 13. Internal tubular organ as Fig. 17. Furca as Fig. 16	*Megaselia fallobreviseta* Disney
–	Without these features combined	52
52	The S8 lobes, with their bare rounded extremities beyond the most posterior bristles, are pale	53
–	The S8 lobes are conspicuously brown (Fig. 23). (Labrum as Fig. 22)	*Megaselia parnassia* Disney
53	Abdominal tergite 6 clearly broader than long. Arista pale (as palp). Furca absent	Species, 25
–	The greatest breadth and length of T6 subequal. Arista brown. A long, heavily sclerotised, furca present	Species, 29
54	Notopleuron with three bristles (or rarely four)	55
–	Notopleuron with only two bristles	60
55	Cerci short (at most 1.6 times as long as greatest breadth, e.g. Figs 5 and 28)	56
–	Cerci longer (at least twice as long as wide)	57
56	Hairs below basal half of hind femur longer than those of the anteroventral row in outer half. A single lobe with many hairs at rear of abdominal sternum 8 (Fig. 6)	*Megaselia kozlovi* sp. n.
–	Hairs below basal half of hind femur shorter than those of the anteroventral row in outer half. A pair of lobes at rear of S8	Species, 18
	Note. This is one possibility for the undescribed female of *Megaselia crellini* Disney, but it is more likely the poorly diagnosed female of *Megaselia nudiventris* (Wood)	
57	Hind femora brown, even if a little paler in basal halves. Postpedicels and labrum brown	58
–	Hind femora yellow with brown tips. Postpedicels yellowish brown. Labrum straw yellow	Species, 3
	Note. This is another possibility for the undescribed female of *Megaselia crellini* Disney	
58	Knob of haltere yellow	59
–	Knob of haltere dark brown. (T6 longer than greatest breadth)	Species, 31
59	Abdominal venter dark grey. Tergite 6 longer than T5. Cerci light brown	Species, 40
–	Abdominal venter light grey. T6 at most as long as T5. Cerci whitish yellow	Species, 27
	Note. If neither applies and hairs below basal half of hind femur are shorter than those of the anteroventral hairs of outer half try couplet 50, lead 2.	
60	Haltere knob yellow. Postpedicels lack SPS vesicles. Hind femur yellow with brown tip and hairs below basal half of hind femur are longer than those of the anteroventral hairs of outer half. With a strongly sclerotised furca (Fig. 26). (Lobes at rear of sternum 8 as Fig. 27. Costal index less than 0.45 and costal cilia less than 0.10 mm long)	Species, 17
–	Without this combination	61
	Note. If it agrees except it lacks such a furca return to couplet 50, as *Megaselia haraldlundi* sometimes has its anterior scutellars only 0.6 times as long as the posterior pair.	
61	Haltere knob brown. Postpedicels lack SPS vesicles	62
–	Haltere knob yellow. Postpedicels with SPS vesicles	63
62	Palps and labrum brown and cerci pale brown. (T6 with maximum width greater than length. Venter light grey. Front femora yellowish brown)	Species, 10
–	Palps, labrum and cerci pale yellow (Upper supra-antennal bristles further apart than pre-ocellar bristles)	Species, 33
63	Cercus less than 3 times as long as broad. Apical bristles of palp longer than glossa	64
–	Cercus more than 4 times as long as broad. Apical bristles of palp shorter than glossa	*Megaselia immodensior* Disney
	Note. The unknown female of *Megaselia elenae* sp. n. will run to this lead.	
64	At least mid and hind femora brown. Wing length more than 1.8 mm. Costal cilia more than 0.13 mm long. Lobes at rear of abdominal sternum 8 with 3 bristles	Species, 2
–	All femora yellow. Wing length less than 1.5 mm and costal cilia less than 0.12 mm long. Lobes at rear of S8 with only 2 bristles	Species, 47

**Figure 10.**
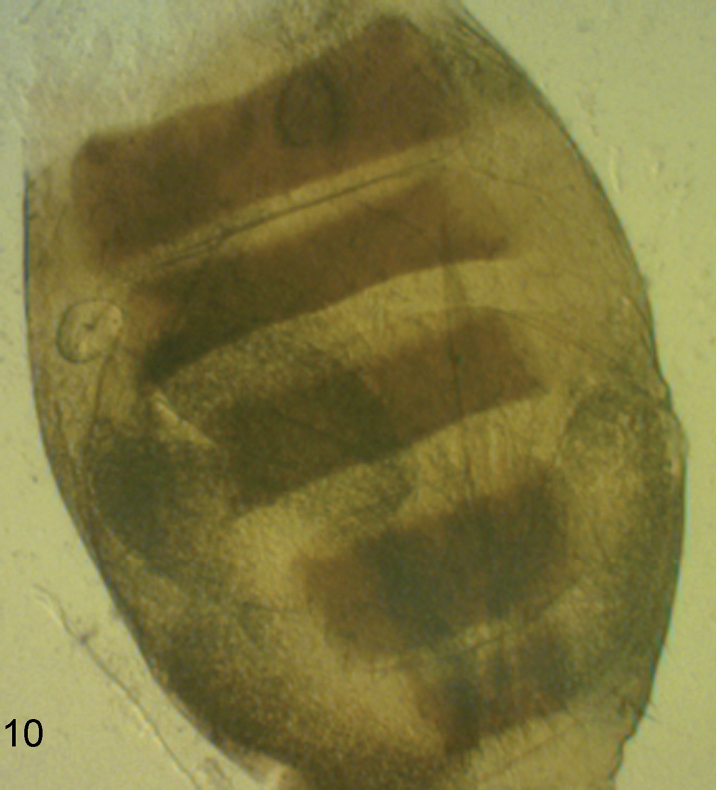
*Megaselia breviterga* female, abdominal tergites 2–6.

**Figure 11.**
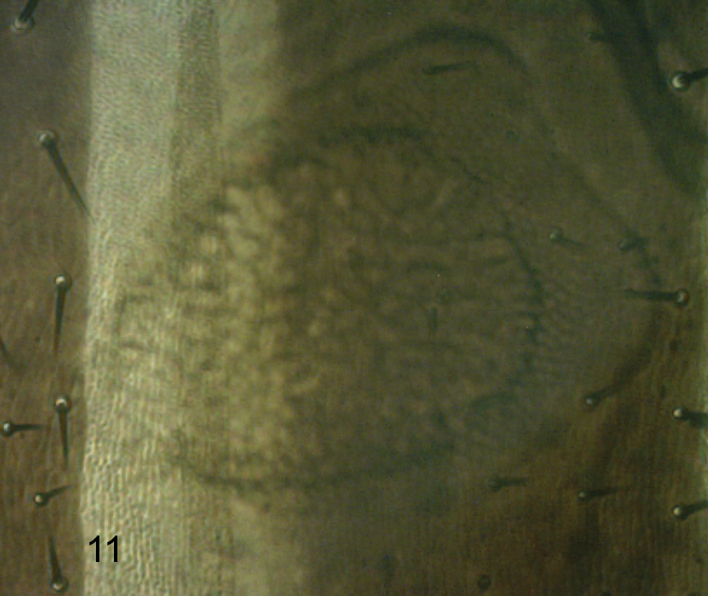
*Megaselia breviterga* female, Dufour’s crop mechanism (anterior end to left).

**Figure 12.**
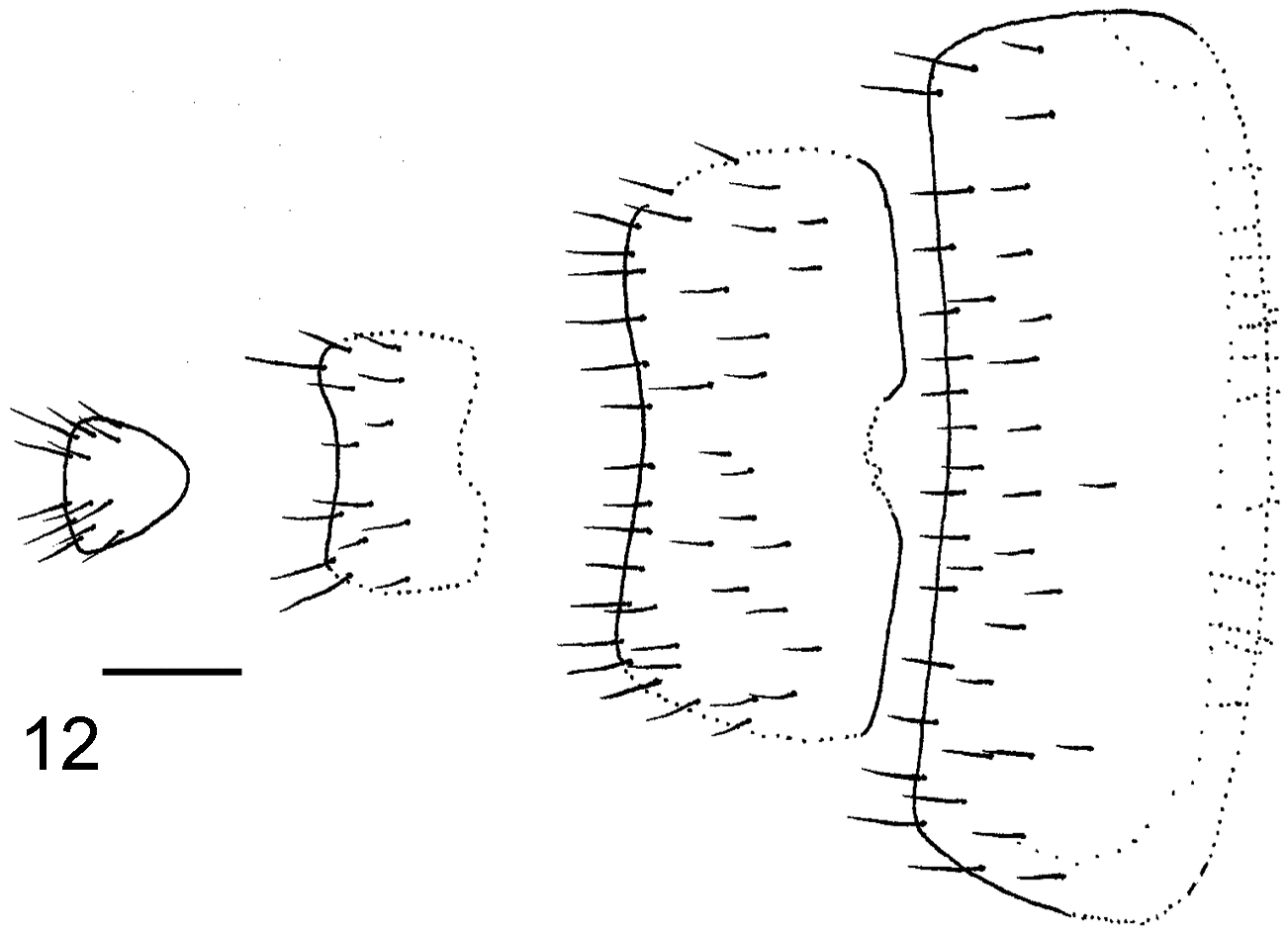
*Megaselia cirriventris* female, abdominal tergites 5–8.

**Figures 13–17.**
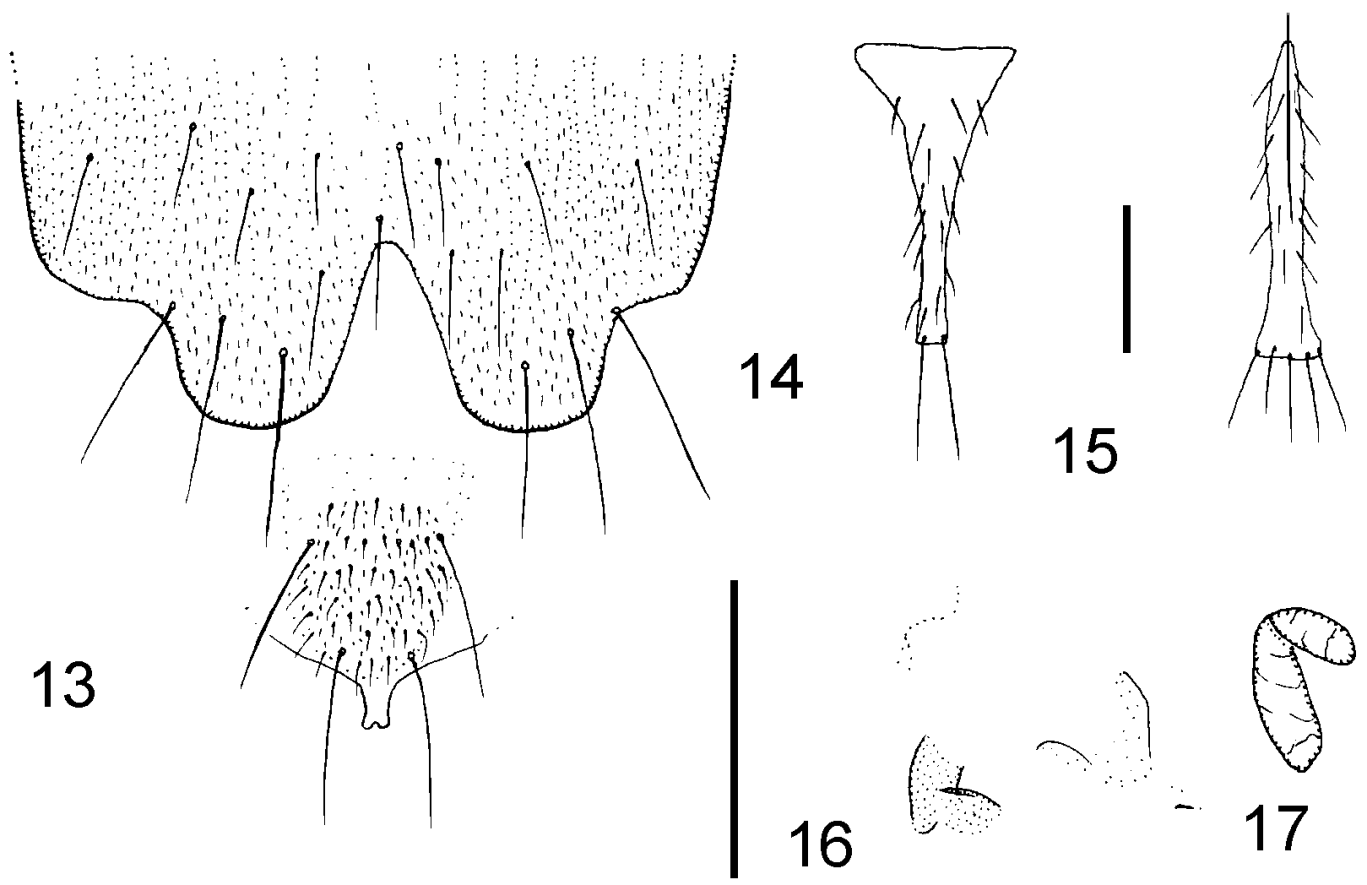
*Megaselia fallobreviseta* female, details of abdomen. **13** rear of sternum 8 and hypoproct **14** tergite 7 **15** sternite 7 **16** furca **17** tubular organ. Scale lines: 0.1mm.

**Figure 18.**
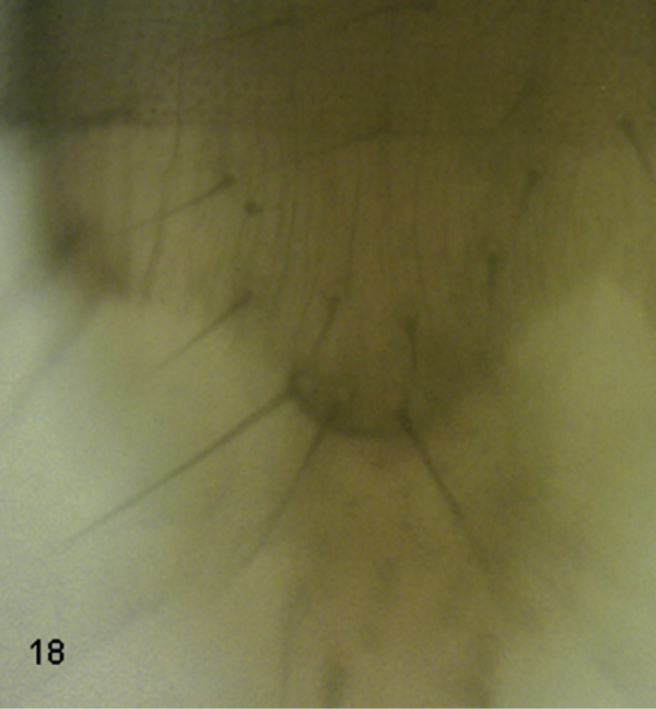
*Megaselia haraldlundi* female, left lobe at rear of abdominal sternum 8.

**Figures 19–20.**
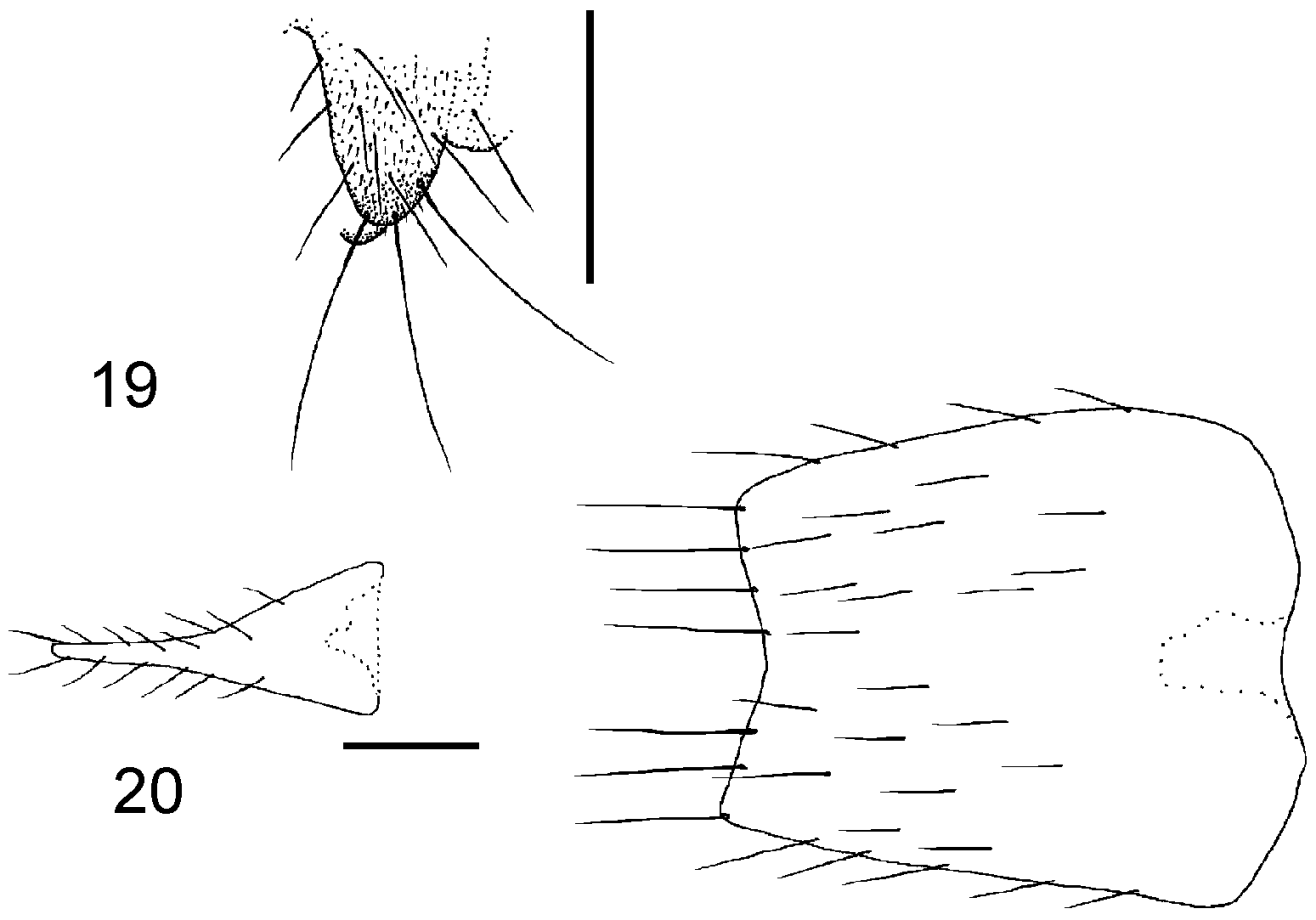
*Megaselia humeralis* female details of abdomen. **19** right lobe at rear of sternum 8 **20** tergites 6 and 7.

**Figure 21.**
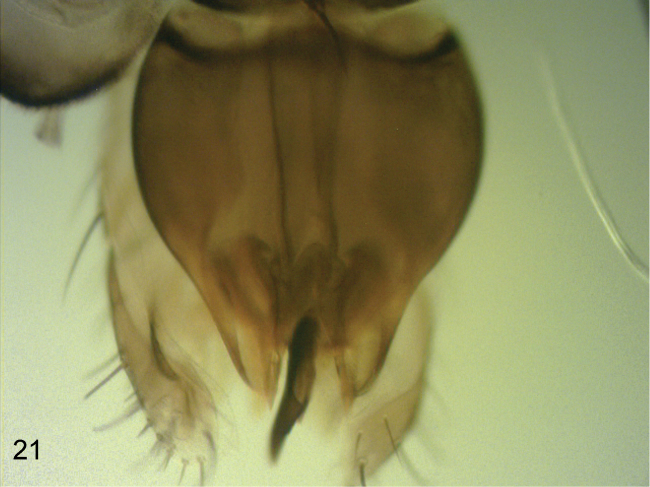
*Megaselia humeralis* female, labrum.

**Figure 22.**
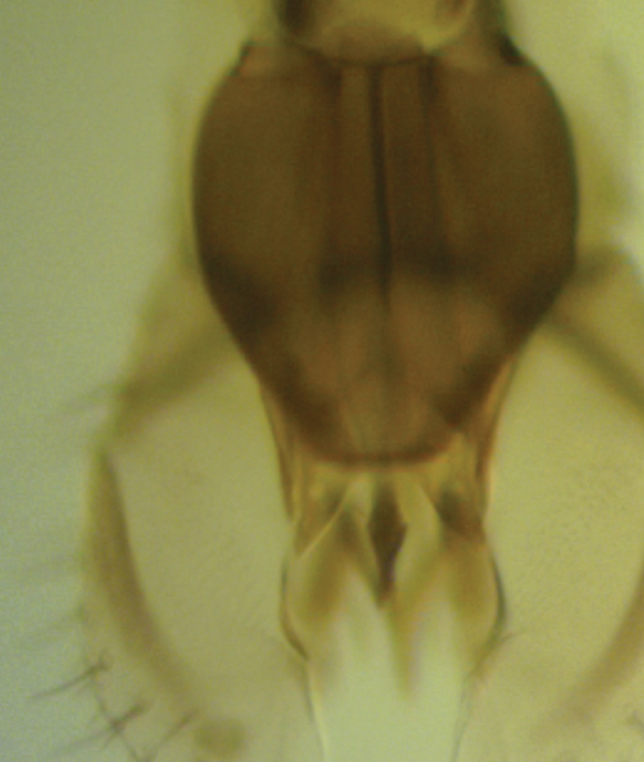
*Megaselia parnassia* female, labrum.

**Figure 23.**
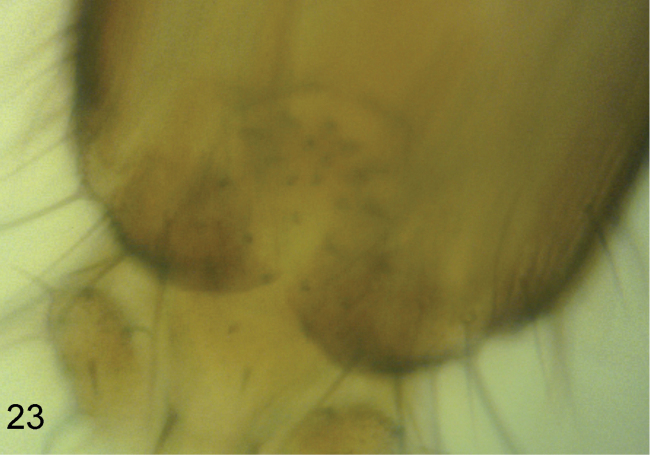
*Megaselia parnassia* female, lobes at rear of abdominal sternum 8.

**Figure 24.**
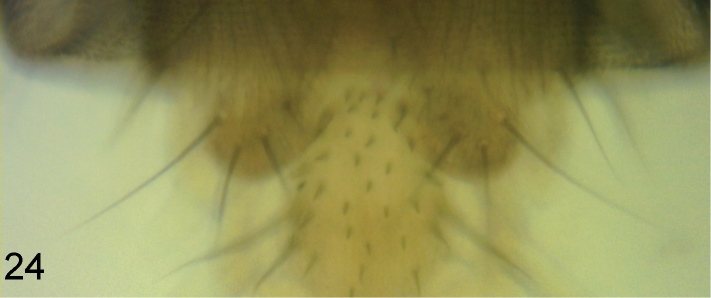
*Megaselia* Species 6, female, lobes at rear of abdominal sternum 8.

**Figure 25.**
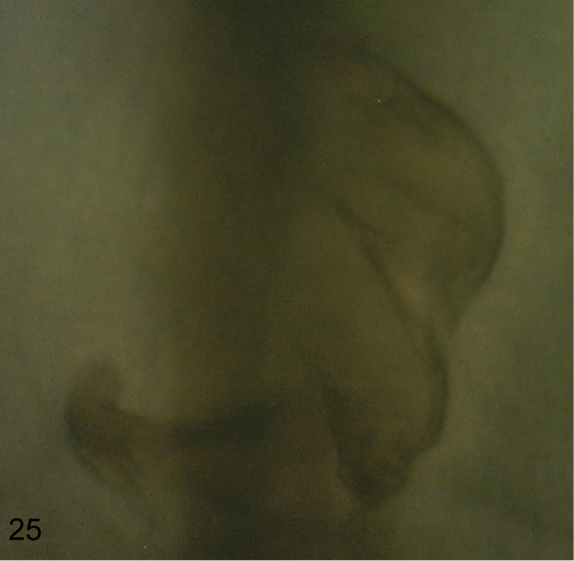
*Megaselia* Species 6, female, furca.

**Figure 26.**
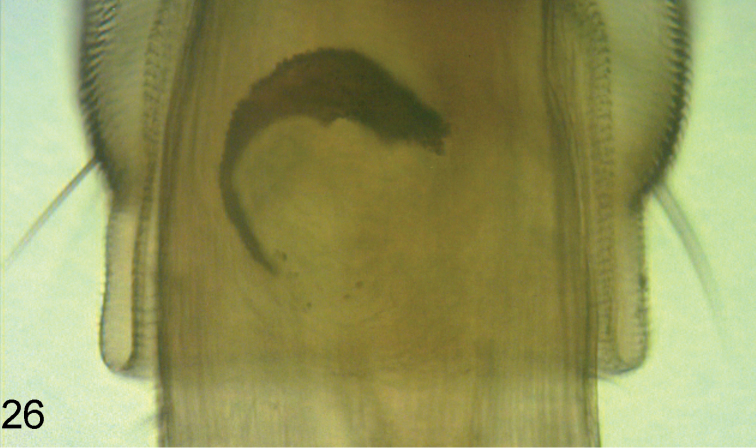
*Megaselia* Species 17, female, furca.

**Figure 27.**
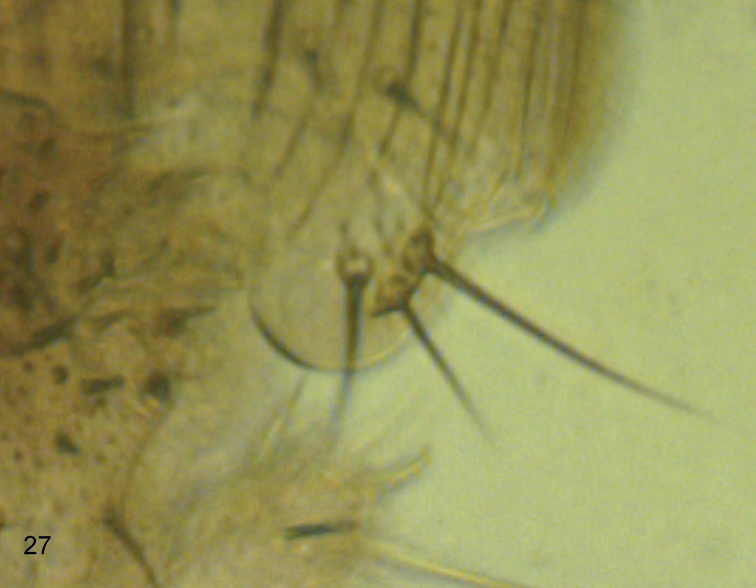
*Megaselia* Species 17, female, left lobe at rear of abdominal sternum 8.

**Figure 28.**
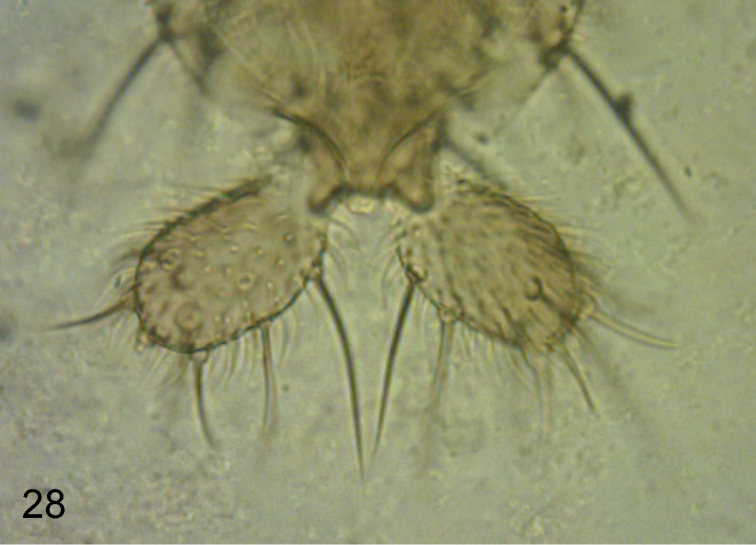
*Megaselia* Species 18, female, cerci from above.

### Key to the males of European species of *Megaselia* with a notopleural cleft

**Figure 29.**
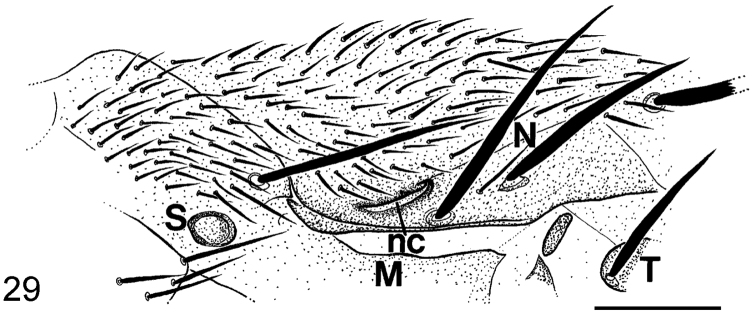
*Megaselia giraudii* male, left notopleuron (nc = notopleural cleft M= mesopleuron, n = notopleural bristles S= anterior spiracle T = tegula at base of wing).

Fig. 29 depicts the notopleural cleft of *Megaselia giraudii*. Reference to most figures are in D (Disney 1989) or in B (Buck and Disney 2001) unless indicated otherwise.

**Table:** 

1	Thorax brown	2
–	Thorax yellow. (Hypopygium as B figs 15 and 16. Hind femur yellow with brown tip)	*Megaselia hexanophila* Buck
2	Halteres entirely brown	3
–	Haltere knob pale yellow	5
3	Palps brown and labella with only a few small spinules below	4
–	Palps yellow and labella with numerous, densely crowded, small spinules below. (Hypopygium as D fig. 380)	*Megaselia hendersoni* Disney
4	Hypopygium as D fig. 494. Costa clearly less than half wing length. Postpedicels lack SPS vesicles	*Megaselia subnudipennis* (Schmitz)
–	Hypopygium as Fig. 30. Costa about half wing length. Postpedicels with SPS vesicles	*Megaselia prodroma* (Lundbeck)
5	Hind femora brown	6
–	Hind femora yellow with brown tips. (Hypopygium as D fig. 393. Palps with short bristles and as B fig. 1)	*Megaselia malhamensis* Disney
	Note: *Megaselia intermedia* (Santos Abréu), only known from the Canary Islands, will run down here. Its hypopygium (fig. 37 in Disney et al. 2010) will immediately distinguish it from *Megaselia malhamensis*.	
6	Postpedicels with SPS vesicles	7
–	Postpedicels without SPS vesicles. (Hypopygium as D fig. 437. Upper supra-antennal bristles clearly wider apart than pre-ocellars (D fig. 432. Palps brown) Front coxae partly yellow)	*Megaselia minuta* (Aldrich)
7	Labella densely spinose below (each with more than 80 small spinules). Postpedicels with at most a dozen SPS vesicles	8
–	Labella with many fewer spinules (less than 50 on each). Postpedicels more than a dozen SPS vesicles	9
8	Notopleural cleft ends behind where it encounters a curved dorsoventral ridge (as Fig. 29). Hypopygium as D fig. 397, each circus having more than 10 hairs	*Megaselia albicans* (Wood)
–	Notopleural cleft does not end behind by encountering such a ridge. Hypopygium as B fig. 19, each cercus having fewer than 10 hairs	*Megaselia offuscata* (Schmitz)
	Note. The unknown male of *Megaselia septentrionalis* may key out here, if it has a notopleural cleft.	
9	Cerci at least twice as long as broad (B fig. 17 and D fig. 400). Wing with at least 3 axillary bristles and costal cilia (of section 3) more than 0.1 mm long	10
–	Cerci clearly less than twice as long as broad (D fig. 446). With only 2 axillary bristles and costal cilia less than 0.1 mm long	*Megaselia brevicostalis* (Wood)
10	Left lobe of hypandrium with numerous microtrichia (D figs 400 and B 17)	11
–	Left lobe of hypandrium lacks microtrichia	*Megaselia elenae* sp. n.
11	Hypopygium as D fig. 400, the left hypandrial lobe being tinged brown and the microtrichia below proctiger tend to be recurved forwards	*Megaselia parnassia* Disney
–	Hypopygium as B fig. 17, the left hypandrial lobe being pale and the microtrichia below proctiger being semi-erect and pointing rearwards	*Megaselia giraudii* (Egger)

**Figure 30.**
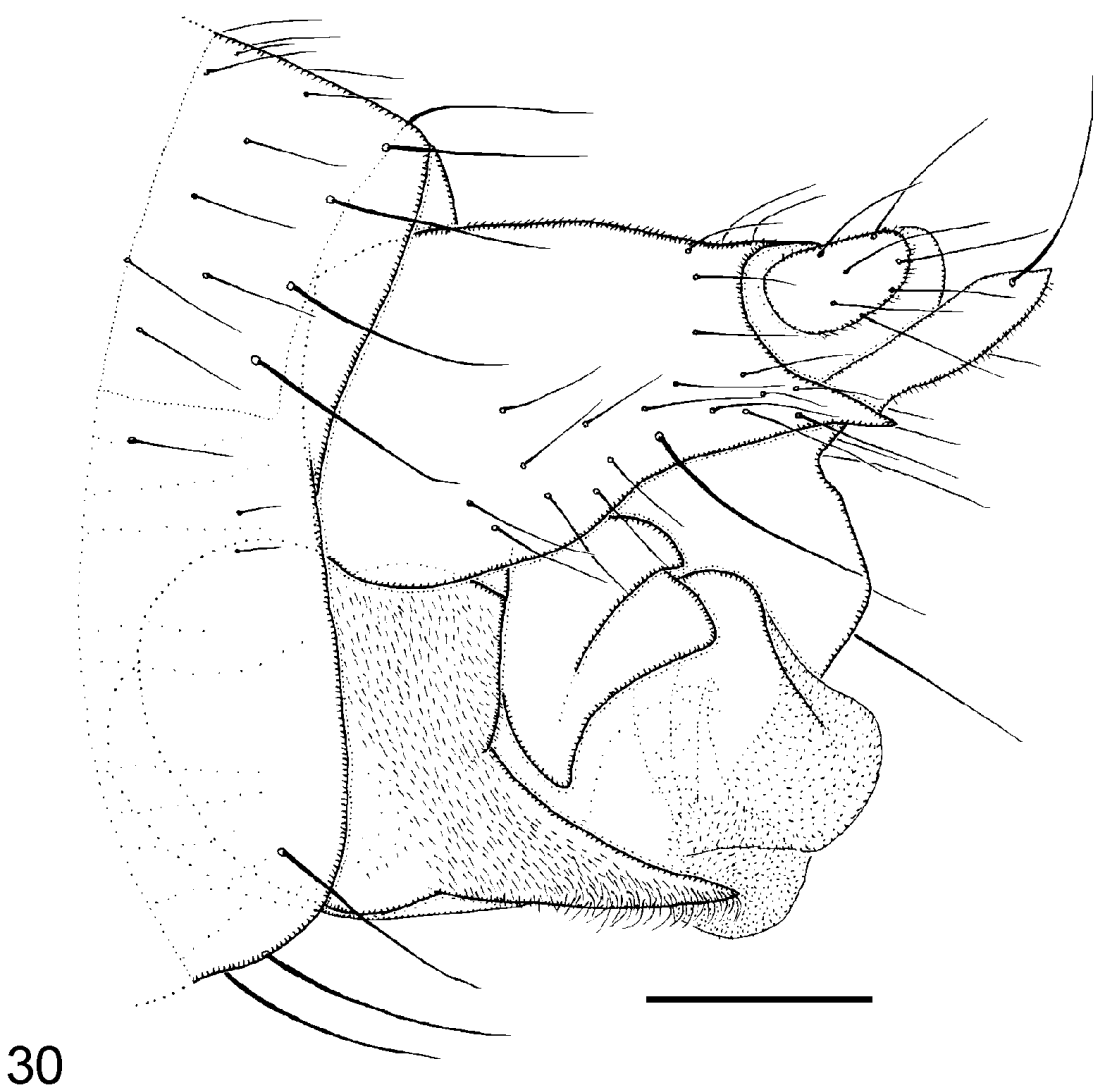
*Megaselia prodroma* male, left face of hypopygium. Scale line: 0.1 mm.

### Tentative key to *Phora* females of species recorded in the Kola Peninsula

**Table:** 

1	Basal half of mid tibia with two anterior bristles. Wing length more than 2.4 mm	2
–	Basal half of mid tibia with only one anterior bristle (very rarely with none).Wing length less than 2.4 mm	4
2	Hind tibia with a single anterodorsal bristle in basal half	3
–	At least one hind tibia with two anterodoral bristles in basal half	*Phora dubia* (Zetterstedt)
3	Costal cilia at level of tip of vein 1 at most two thirds the length of axillary bristles. Segments 2–5 of front tarsus relatively stout	*Phora stictica* Meigen
–	Costal cilia at level of tip of vein 1 at least four fifths the length of axillary bristles. Segments 2–5 of front tarsus not so stout	*Phora artifrons* Schmitz
4	The separation of the females of *Phora pubipes* Schmitzand *Phora holosericea* Schmitz has been based on minute differences in the distribution of microtrichia on the hind trochanters and microsculpture on the hind femora. However, there are probably better differences in the proboscis. At present I only have two poor females of *Phora holosericea* that were procured mating with males. Until better voucher specimens of both species, and other species of *Phora*, are available for both these species reliable differences cannot be proposed with any confidence. Apart from mating pairs, reared series or specimens for which molecular barccodes have been determined are required.	

## Discussion

The number species recorded above is far larger than expected. Only six species are known to occur in Greenland, with only three established north of the Arctic Circle (Disney, in press). For Iceland, which lies immediately south of the Arctic Circle, 11 species have been recorded (Prescher et al. 2005). For the British Isles more than 340 species have been recorded so far, with at least 80 of these being recorded in my suburban garden in Cambridge. In the Antarctic one species being accidentally introduced by man has been reported (Nickolls and Disney 2001) and a second species introduced by man has become established on islands in the South Atlantic Ocean to the north of the Antarctic Circle (Hänel and Disney 2006, Jones et al. 2003). It would seem that the impoverished faunas of these situations is due more to their remoteness than to their high latitudes. By contrast the Kola Peninsula is attached to the mainland of Europe.

## Supplementary Material

XML Treatment for Megaselia elenae

XML Treatment for Megaselia kozlovi
